# Electroless plating of premetalized polyamide fibers for stretchable conductive devices†

**DOI:** 10.1039/d3ra01566a

**Published:** 2023-06-20

**Authors:** P. Vishakha T. Weerasinghe, Ruchira N. Wijesena, Nadeeka D. Tissera, Gayan Priyadarshana, Nandula D. Wanasekara, D. G. Kanchana Dissanayake, K. M. Nalin de Silva

**Affiliations:** a Department of Textile and Clothing Textile and Clothing Technology, University of Moratuwa Moratuwa Sri Lanka; b Division of Textile and Clothing Technology, Institute of Technology, University of Moratuwa Diyagama Homagama Sri Lanka ruchiraw@itum.mrt.ac.lk; c Department of Chemistry, University of Colombo Colombo 03 Sri Lanka kmnd@chem.cmb.ac.lk; d Department of Engineering Technology, Faculty of Technology, University of Sri Jayewardenepura Gangodawila Nugegoda Sri Lanka

## Abstract

A new approach was used to produce electrically conductive polyamide yarns, employing an electroless plating technique, which involved stabilizing silver nanoparticles on the surface of the yarn using Sn^2+^. First, the [Ag(NH_3_)^2^]^+^ complex was reduced using Sn^2+^ to produce silver nanoparticle seed layers on the fiber surface, followed by a formaldehyde reduction. The nucleation and growth of silver nanoparticles on the fiber surface were observed through SEM images, demonstrating varying degrees of silver deposition depending on the silver concentration. This deposition variation was confirmed through XRD patterns, TGA data and UV-vis spectra. Additionally, XPS characterization showed the evolution of the chemical state of silver and tin during the silver reduction process. Electrical resistance revealed that the resistance per unit length of the yarn ranged from 3 ± 0.3 Ω cm^−1^ to 70 ± 6 Ω cm^−1^, depending on the silver concentration. The resulting silver-plated yarn was incorporated into a stretchable device, demonstrating stable resistance over multiple cycles. This method of fabricating conductive yarn has the potential to open up new possibilities in the design and manufacture of stretchable conductive devices for flexible electronics.

## Abstract 2

A new approach was used to produce electrically conductive polyamide yarns, employing an electroless plating technique, which involved stabilizing silver nanoparticles on the surface of the yarn using Sn^2+^.

## Graphical abstract

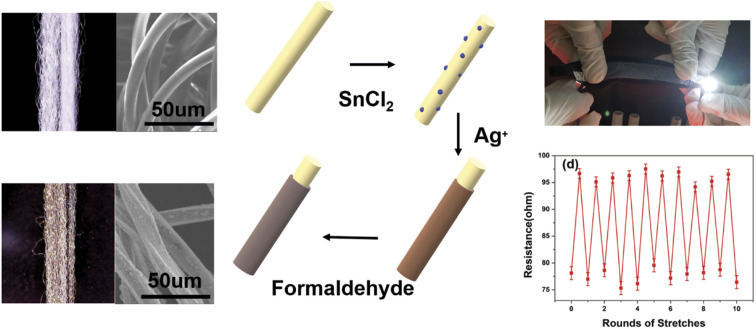

## Introduction

The outstanding properties of wearable/flexible electronics, such as lightweight, flexibility, softness, and comfort, have attracted significant interest in recent years. Smart textiles, or e-textiles, have emerged as a promising area of research due to the potential to integrate electronics into fibers, yarns, fabrics, or garments.^1^

Textile materials are often used in close proximity to the human body, making them an attractive platform for smart applications. Some of the applications that are already being researched include textile based sensors/detectors, knitted switches, embroidered antennas, inter-connected lines and other wearable/flexible electronics applications.^2–4^ However, conventional textile materials are usually electrically insulating, which limits their potential for use in smart wearable/flexible electronics applications. To overcome this limitation, several processes have been developed to introduce a conductive material onto or into textile materials.

However, these methods, including sputtering, chemical vapor deposition (CVD),^5^ physical vapor deposition (PVD),^6^ ultrasound irradiation,^7^ and sonochemical deposition,^8^ require high energy, high temperature treatment, and vacuum conditions, which limit their widespread adaptability due to process incompatibility and high cost. Alternatively electroplating approach can be adapted, but it requires a conductive substrate.^9^ Therefore, it is not appropriate for non-conductive textile materials such as polyamide, polyester, cotton and *etc.* Careful investigation of these techniques reveals that there is a lot of space to explore to make these processes more efficient and to introduce novelty. This will surely help mitigate some of the current challenges in these materials making them further available to the public.

Electroless plating can be considered as a much more practical approach in preparation of metallic conductive fibers, in which metal irons are reduced to metal particles onto a surface from a metal salt solution without the use of an electrical potential.^10^ Therefore, the surface does not require to be electronically conducting, while the kinetics of electron transfer must be slow enough to hold off the reduction of the metal ions and nucleation in solution. Thus, electroless plating can be introduced as a versatile process, because of its uniform coating, low cost and can be done over non-conductive substrate.^10^

Silver plated fibers and fabrics are of highest interest, due to the high electrical conductive properties, anti-microbial activities, stability against oxidation and, relatively low toxicity to human cells [9]. More recently, reports of production of electroless plating of silver are based on *in situ* synthesis of nano-silver on nylon fibers treated with potassium permanganate,^11^ silver reduction on chitosan coated lead (Pd) incorporated aramid fibers,^12^ reduction of silver complex directly on polyamide fibers without using any reducing agents.^13^ Polyamide 6 (nylon 6) is commercially available due to its excellent properties.^11,14,15^ Furthermore, nylon has been selected as the substrate in order to obtain a high improvement of adhesion of silver layer, because it is a comparatively polar polymer synthesized by adipoyl chlorid and hexamethylene diamine monomers and has electronic rich linear molecular chain.^16^

Although different methodologies have been used to achieve silver electroless plated fibers, in this paper, we investigated the preparation of a conductive silver layer on the fabric surface based on a common approach to deposit metals on surfaces. This method has recently been used for electroless silver plating silica spheres,^17^ polystyrene,^18^ carbon nanotubes,^19^ carbon nanofibers,^15^ but has not been implemented on polyamide fibers until now. Silver layer has been synthesized on the fabric surface by a two-step chemical reduction process involving Sn^2+^ ions and formaldehyde. The surface morphology, electrical properties and material properties of polyester fibers were investigated. Applicability of the yarn for stretchable conductive device was also demonstrated. The results are thoroughly discussed in this article.

## Experimental

### Materials and reagents

1/42 Dtex 48F polyamide 6 filaments were obtained from Teejay Lanka PLC. Silver nitrate (AgNO_3_) ACS reagent, ≥99.0% and formaldehyde solution, meets analytical specification of USP, ≥34.5 wt% was supplied from Sigma-Aldrich, ammonia solution extrapure AR, 25% and concentrated hydrochloric acid (HCl, 37%) extrapure AR was purchased from Sisco Research Laboratories Pvt. Ltd, tin powder (Sn), ethylenediaminetetraacetic acid (EDTA-Na_2_) and liquid silicone rubber was obtained by Sri Lanka Institute of Nanotechnology.

### Synthesizing of silver nanoparticles on the knitted fabric

Sn powder was dissolved in HCl (37%) to prepare a 3 g per mL SnCl_2_ solution, then 37% concentrated excess HCL in the solution was diluted to 10%. Nylon-6 filament yarn was knitted into a tubular single jersey 200 g m^−2^ fabric sample and scoured (see Section S1 of ESI†). This scoured fabric sample was labelled as ‘polyamide’ during characterization. The fabric sample was immersed in 2 mL of the solution SnCl_2_, stirring for 3 min and rinsed with distilled water. The fabric sample was stirred in a 10 mL of 0.366 g per L EDTA-Na_2_ solution. Ag[(NH_3_)]^2+^ complex was added dropwise to the bath contained the fabric sample and stirred until the solution got clear and fabric color changed to brown. This was followed by addition of formaldehyde and stirred for 3 hours. Finally, goldish gray color fabric sample was rinsed with distilled water and dried. To achieve different degrees of silver plating, different amounts of silver concentrations were used as given in the Table 1. In addition to these four samples, a sample was prepared during Ag 5% sample preparation procedure without the formaldehyde reduction (WFR) procedure, this was labelled ‘WFR’.

**Table 1:** AgNO_3_ concentrations, ammonia volumes used to prepare clear Tollen's regent and formaldehyde volumes to reduce excess Ag^+^ on silver seeds which are reduced by Sn^2+^

Sample ref	AgNO_3_ weight (g) in volume of 3 mL, concentration (w/v %)	Ammonia solution (μL)	Formaldehyde (μL)
Ag – 2.5%	0.08, 2.5	88	45
Ag – 5%	0.15, 5	176	90
Ag – 10%	0.30, 10	352	180
Ag – 20%	0.61, 20	702	360
The sample prepared without formaldehyde	0.15, 5	176	—

### Physicochemical characterization

Electrical resistance measurements were performed on yarn samples unravelled from silver plated fabric samples employing a multimeter (73204-Yokogawa Digital Multimeter). Resistance was measured along the yarn between 5 cm distance and average was taken from 10 measurements. Ultimately, resistance per unit length was calculated from the formula given in eqn (1), where *R* and *l* are resistance and length of the yarn, respectively:1
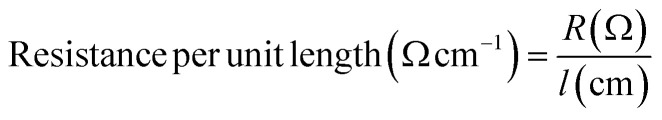


The surface morphology of silver plated polyamide fibers was imaged with a scanning electron microscope (SEM, TM-1000, Hitachi). X-ray photoelectron spectroscopy (XPS) was performed on a Thermo Scientific Kα XPS to determine the chemical state of both silver and tin. The crystal structure of silver nanoparticles on the polyamide fabrics was illustrated by X-ray diffraction (XRD, Brucker D8 Focus X-ray diffractometer) operated at 30 kV and 20 mA. Fourier transform infrared (FT-IR) spectroscopy was performed using Bruker FT-IR Vertex 80 to investigate the functional groups on the surface of the samples. The FT-IR spectra were recorded using attenuated total reflectance (ATR) measurement mode with the resolution of 4 cm^−1^ between 4000 cm^−1^ to 600 cm^−1^, the sample scan time was set as 64 seconds. Shimadzu UV-vis-NIR spectrophotometer was used to obtain the UV visible (UV-vis) reflectance spectra of different samples by irradiation of light in the wavelengths between 300 and 700 nm. The reflectance spectrum data was transferred to absorption data by the use of Kubelka–Munk eqn (2):2
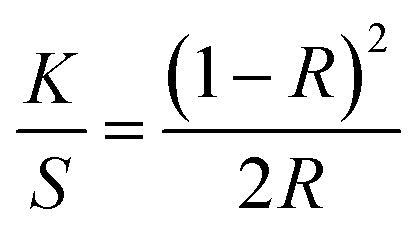
where *K* is the absorption coefficient, *R* is the diffuse reflectance of the silver plated fabric, and *S* is the scattering coefficient.

Were heated from room temperature to 1000 °C at a heating rate of 20 °C min^−1^. The percentage of weight loss and ash content were studied from TGA curves.

A device was fabricated from the developed yarn and investigate the effect of stretching over the resistance of the device (ESI Section S2†).

## Results and discussion

### Acid hydrolysis of nylon fabric and *in situ* preparation of silver layer on the yarn surface to produce polyamide/silver composite

**Fig. 1:**
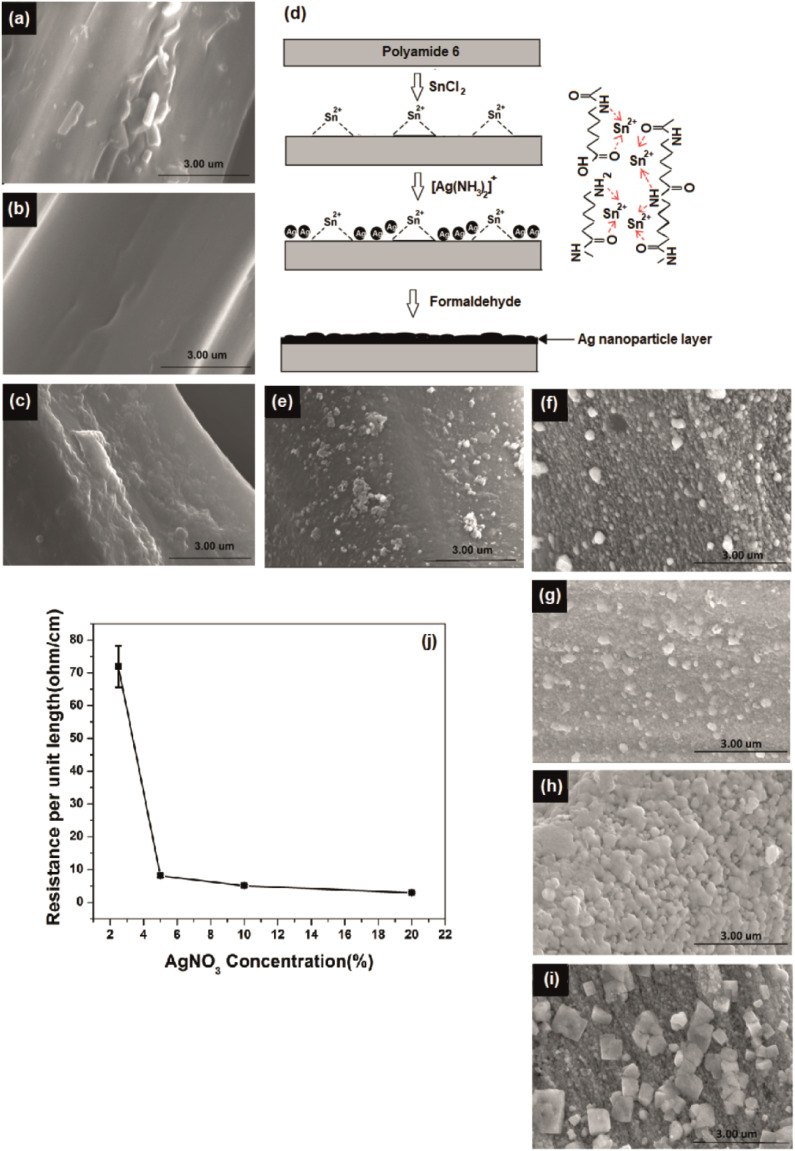
SEM images of polyamide 6 fibers, (a) untreated fibers, (b) fiber impurities removed by scouring, (c) hydrolysed fiber surface. (d) Proposed mechanism of electroless plating. (e–i) Silver reduced polyamide surface, (e) WFR, (f) Ag 2.5%, (g) Ag 5%, (h) Ag 10%, (i) Ag 20%. (j) Resistance per unit length verses used AgNO_3_ concentration in each Tollen's reagent to create silver layer on fiber surface by reduction of formaldehyde.

Initially, the nylon fabric was scoured and washed to remove any impurities present on the yarn surface that might affect uniformity and efficiency of plating. The fiber surface before and after scouring is shown in Fig. 1a and b, respectively.

The silver-plating process is implemented by reduction of Ag^+^ to Ag^0^ through oxidation of Sn^2+^ to Sn^4+^.^17^ To introduce Sn^2+^ on polyamide surface, the scoured nylon fabric sample was immersed in a SnCl_2_ solution with additional HCl. Here, HCl in the solution suppresses the hydrolysis of SnCl_2_ to water insoluble salts of Sn(OH)Cl and prevents the oxidation of Sn^2+^ to Sn^4+^.^15,20^ Also, the H^+^ in the solution further hydrolysis the polyamide and generate polar groups such as –NH_2_ and –COOH on the fiber surface. The hydride irons in the SnCl_2_/HCl solution formed ions (–NH_3_^+^) end groups on the fiber surface^14^ (ESI Section S3†) which greatly improved the hydrophilicity.^15,21^ In addition, low molecular weight segments were removed and pits and holes were created in the fiber by these reactions, which increased the surface roughness and surface area of the yarn (SEM Fig. 1c).

These pits and functional groups formed on the fiber surface promote the adsorption of Sn^2+^ on the yarn surface by physical interlocking and chemical bonding. Consequently, the roughened fiber surface obstructs the detachment of the final silver layer and contributes to a high interfacial adhesion, known as the anchoring effect.^22^ The silver iron bath is unstable due to the self-catalysing effect of silver. Owing to its high redox potential silver is able to reduce easily and form agglomerates. Therefore, silver atoms can be deposit in solution,^23^ resulting in insufficient growth on the polyamide surface during electroless deposition. To ensure successful and uniform deposition of silver particles on the fiber surface, the fiber surface was activated with Sn^2+^ as a reducing agent. The schematic diagram in Fig. 1d indicates the possible interactions between polyamide and Sn^2+^.

### Preparation of polyamide/silver composite

Several methods have been utilized to form silver nanoparticles. These synthesis methods were based on photoreduction; chemical reduction; Tollen's reagent; and sol–gel, polyol, and biochemical approaches.^24–29^ Sn^2+^ is a commonly used reducing agent for synthesizing nanoparticles from Tollen's^29^ reagent through a redox reaction on the surface that resulted from oxidation of Sn^2+^ to Sn^4+^ and reduction of Ag^+^ to Ag^0^ (refs. ^18^ and 30) (see ESI Section S4†).

The addition of EDTA-Na_2_ solution leads to perform as an additional complexing agent for silver with has the capability to preserve the silver ion particles in the solution for a longer period of time by preventing the agglomeration of silver particles in the solution.^31^ This was followed by Tollen's reagent, as a result, a redox reaction on the surface that resulted in oxidation of Sn^2+^ to Sn^4+^ and reduction of Ag^+^ to Ag^0^ given by the eqn (3) and (4):

3 Sn^2+^ → Sn^4+^ + 2e

4 2Ag^+^ + 2e → 2Ag↓

Further, formaldehyde was added to the solution to obtain continuous growth of the silver seeds and silver nanoparticles connected to each other that led to formation of a high-density silver layer which is highly conductive (Fig. 1d). Fig. S2† illustrates that formed particles are nano-size particles. The reaction between formaldehyde and silver ions in alkali solution^32^ is given by the eqn (5) and (6):

5 2Ag^+^ + HCHO + 3OH^−^ → 2Ag + HCOO^−^ + 2H_2_O

6
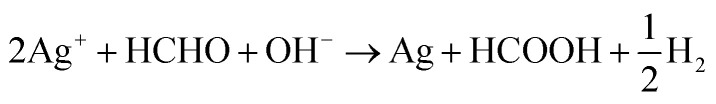
Here, hydride and formate irons were produced by nucleophilic addition reaction of formaldehyde by hydroxyl groups. Silver irons were reduced to silver atoms with production of hydrogen as a by-product by hydride ions.

### Influence of silver concentration on the electrical resistance

The AgNO_3_ concentration was varied and NH_4_OH was added according to the silver concentrations to obtain clear solutions of Tollen's reagent. The morphology of the fiber surface was studied for different AgNO_3_ concentrations, as shown in Fig. 1e–i. The degree of deposition increased with increasing AgNO_3_ concentration. The effect of Ag^+^ concentration on reducing the electrical resistance of the silver yarns is shown in Fig. 1j. Electroless silver plating without formaldehyde reduction resulted in the deposition of silver nanoparticles on the fiber surface (Fig. 1d). However, this sample has a low degree of deposition with discontinuities, which was insufficient to create a conductive path on the fiber surface. As a result, the yarns were not conductive without formaldehyde reduction. Upon formaldehyde reduction, a significant amount of silver ions was reduced on the fiber surface, as demonstrated in Fig. 1j. By increasing the concentration of silver from 2.5% to 20% through formaldehyde reduction, we were able to achieve a significant reduction in resistance, from 71.9 ± 5 to 2.93 ± 0.2 Ω cm^−1^.

### Chemical and structure analysis of silver deposition

**Fig. 2:**
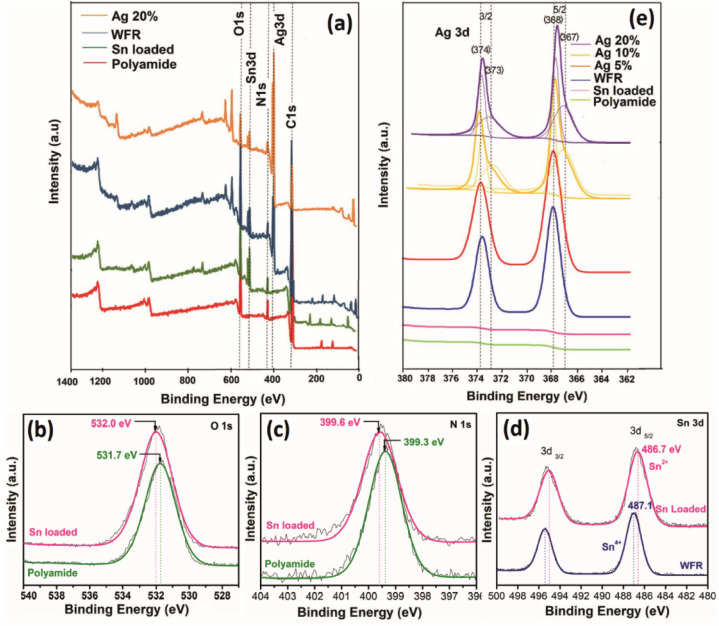
X-ray photoelectron spectra (a) wide scan spectra of fabric samples at different stages of the process, high resolution spectra of (b) O 1s, (c) N 1s, (d) Sn 3d and (e) Ag 3d.

XPS analysis was performed on silver-plated polyamide fabric samples to investigate the details of the chemical state of the silver-plating process. The results of the surface element analyses are reported in Fig. 2. Characteristic lines are illustrated in Fig. 2a as C 1s, Ag 3d, N 1s, Sn 3d and O 1s at binding energy values around 284, 368, 399, 487 and 531 eV, respectively.^16,33,34^ In addition, other impurities were not detected in the range up to 1400 eV. As can be clearly seen from the wide range XPS spectra in Fig. 2a, the spectrum of polyamide shows the characteristic peaks for C, N, and O, as expected, while the polyamide fabric loaded with Sn^2+^ shows an additional peak for Sn, indicating the presence of Sn^2+^ on the surface of the polyamide fabric after the treatment of SnCl_2_/HCl. Moreover, the appearance of a new peak for Ag can be seen for WFR and Ag 20% samples while the rest of the peaks remained unchanged. It is worth noticing that, during the deposition of Ag, the Ag^+^ ions were reduced by Sn^2+^ (WFR) to form Ag. Further addition of formaldehyde (in Ag 20%) leads to nucleation and growth of Ag nanoparticles, resulting in a perfect coating of Ag nanoparticles on the surface of the fabric. This argument is supported by the XPS data obtained for WFR and Ag 20%, in which the relative peak intensity of Ag 3d increased from WFR to Ag 20%, while that of Sn 3d showed a decrease in peak intensity (see Fig. 2a).

High-resolution XPS spectra of O 1s and N 1s obtained for the polyamide fabric before and after HCl/SnCl_2_ treatment are shown in Fig. 2b and c. As can be clearly seen in these figures, both the O 1s and N 1s peaks are shifted toward higher binding energy values, indicating a possible ionic interaction between the Sn^2+^ ions and C=O/NH_2_ functional groups in the polyamide backbone. The Sn^2+^ ions could react with Lewis base sites and negatively charged functional groups of amide and acid end groups as well as amine end groups (formed after hydrolysis of amide). This has already been confirmed in the literature^11,35^ and these bonds ensure that the reduction of Sn^2+^ ions take place only at the fiber surface and not in the solution, while the carbonyl and amine groups act as catalysts so that the remaining ions are bound. The schematic diagram in Fig. 1d shows the possible interactions between polyamide and Sn^2+^ validated from the high-resolution XPS data. When the silver content was increased, Ag 3d 3/2 and 5/2 were further divided into four peaks confirming the presence of both Ag^+^ and Ag^0^ species at about 367.9/368.6 eV (Ag^+^/Ag^0^) and 373.9/374.6 eV (Ag^+^/Ag^0^)^35,36^ (Fig. 2e). This could be due to the absorption of excess Ag^+^ species in carbonyl and amine groups on the polyamide chains by ionic interaction, which could not be reduced by the available amount of Sn^2+^.^11^ Also these adsorbed [Ag(NH_3_)_2_]^+^ complexing ions could slowly release silver ions in the presence of OH^−^ ions. This leads to the formation of AgOH, which can be rapidly decomposed to synthesize the Ag_2_O nucleus^37^ as given in eqn (7) and (8):

7 [Ag(NH_3_)_2_]^+^ + OH^−^ ⇋ AgOH + 2NH_3_

8 2Ag^+^ + HCHO + 3OH^−^ → 2Ag + HCOO^−^ + 2H_2_O

**Fig. 3:**
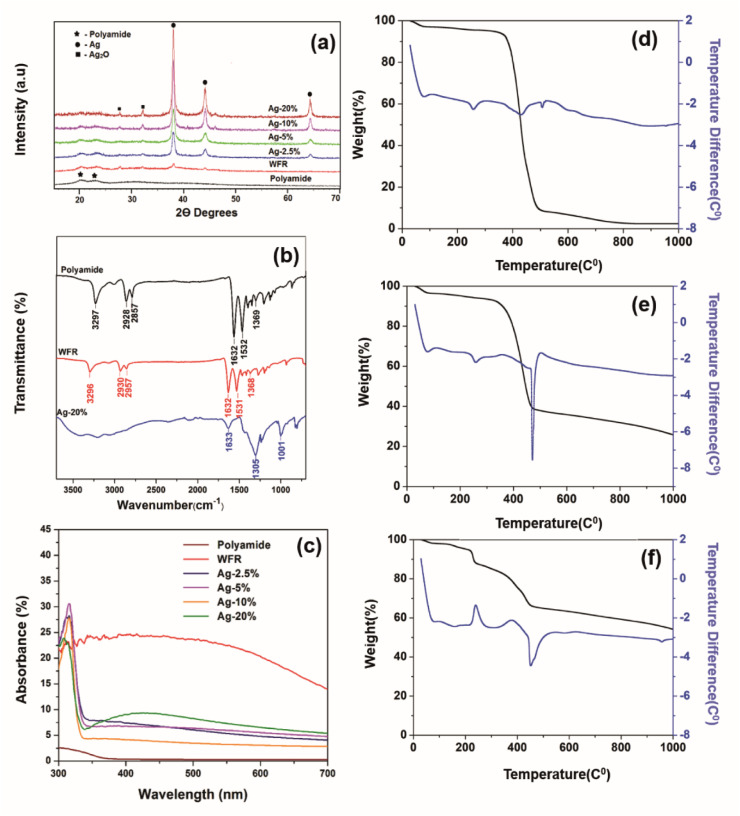
(a) X-ray diffraction pattern, (b) UV-visible spectra, (c) FTIR absorption spectra of TGA thermogram of (d) polyamide, (e) WFR and (f) Ag 20%.

The XRD spectra presented in Fig. 3a has obvious sharp peaks of Ag at 2*θ* of 43.5° and 63.6° corresponding to the (200) and (220).^38^ Comparatively weak XRD peaks appear for Ag_2_O at 26.7°, 32.8° correlate to respective (110) and (111)^39^ and also the strongest peak Ag/Ag_2_O peak at 38.1 attributes to (111) and (200).^40^ Although the Ag peaks are not prominent before formaldehyde reduction, peak intensities were increased after formaldehyde reduction. Moreover, very strong silver peaks have emerged after the increase of the concentration of silver ions in the solution. Also, the silver oxide peaks appear with increasing silver concentration which are indicated clearly at 10% and 20% silver concentrations. The existence of silver oxide that shows peaks corresponding to Ag^+^ due to Ag_2_O in the XPS spectrum of same concentrations in Fig. 2e was confirmed by XRD.^41^

The FTIR spectra of untreated polyamide fibers, a sample before formaldehyde reduction and a sample after formaldehyde reduction are shown in Fig. 3b. The characteristic peaks were attributed to –C–H_2_– stretching at 2928 cm^−1^ and at 2857 cm^−1^, N–H stretching. The absorption peak at 3297 cm^−1^ can be attributed to the stretching vibration of N–H, C=O stretching vibration (amide I) at 1632 cm^−1^, the peak at 1532 cm^−1^ is correlated to N–H deformation and C–N stretching (amide II) and the peak at 1369 cm^−1^ is due to C–H stretching.^42^ After Ag^+^ was reduced to Ag_2_O by Sn^2+^, new peaks do not appear, but there is a shortening in the corresponding peaks of polyamide 6 compared to the control. The reason might have been that chemical and physical interaction of functional groups of polyamide 6 and silver nanoparticles. This becomes more prominent when increasing the silver loading by further reduction of silver irons by formaldehyde as shown in the spectra of Ag 20%.^43^

The absorption was greatly increased (see Fig. 3c), which is due to the formation of silver nanoparticles that have a dark brown color due to the oscillation of free electrons in the silver nanoparticles.^44^ The SEM images illustrate that the silver seeds were formed by further reduction of silver with formaldehyde. Normally, the reduction of silver iron in Tollen's reagent by formaldehyde is used to obtain a silver mirror, but here it was used to apply a silver mirror on the fabric surface. This could lead to higher reflectance, but on the other hand, it could also reduce absorption. Moreover, the images from SEM show the aggregation and growth of silver nanoparticles with increasing silver concentration. The electron density in the conductive band closer to the nanoparticle surface is higher with increasing particle size, therefore the collective excitation leads to surface plasmon resonance, which was shown as plasmonic absorption in the figure.^45^

Themogram of polyamide (Fig. 3d) presents the mass loss starting at about 50 °C and continuing up to 105 °C, which is due to the loss of water molecules.^46^ The mass loss is merely constant from approximately up to 372 °C, then a rapid mass loss is observed at temperatures between 372 and 508 °C, which is due to the loss of oligomers and the continuous mass loss due to the complete decomposition of polyamide.^47^

The rate of degradation of the material becomes low at higher temperatures and some ash residue remains after 800 °C. The weight loss of Ag/polyamide composite samples is comparatively less than that of pure polyamide. By incorporating silver and increasing the dose, a higher residual ash and lower weight loss is observed (Table S1†), which can be attributed to the silver composition of the sample.

### Electrical properties of the stretchable conductive device and knitted fabric

This study shows that the manufacturing process of electroless silver-plated conductive polyamide yarns can be easily extended to produce stretchable conductive devices for smart textile applications.

**Fig. 4:**
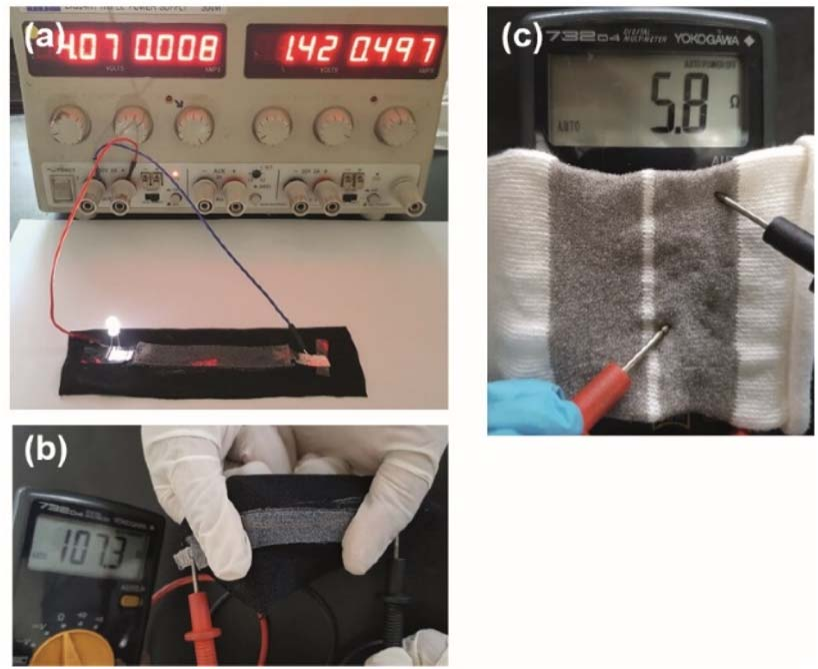
(a) Stretchable conductive device from conductive yarns is connected to the simple circuit to light LED. (b) Measuring the resistance of stretched device. (c) Stretchable conductive device from conductive fabric.

As a proof-of-concept, a stretchable conductive device was prepared by sandwiching a yarn between a fabric and a silicon layer through screen printing. A simple circuit was then constructed by connecting a power supply set at 4 V and a LED lamp to one end of the stretchable conductive device (see Fig. 4a). Upon completion of the circuit, the LED lamp immediately lit up and illuminated the LED until the circuit was disconnected.

Importantly, upon stretch and recovery, the resistance of the device was recorded between 75 and 110 Ω suggesting that silver plated yarn obtained by unravelling the conductive fabric has a high potential of producing stretchable conductive devices with elastomeric materials. Fig. 4b shows the resistance measurements of the device that was stretched.

In addition, the unravelled yarn can be converted into various types of electronic devices such as switches, capacitors and resistors by knitting into various designs. The Fig. 4c shows a knitted capacitor fabricated by knitting the conductive yarn as the two electrodes and polyamide yarn as an electrolyte.

**Fig. 5:**
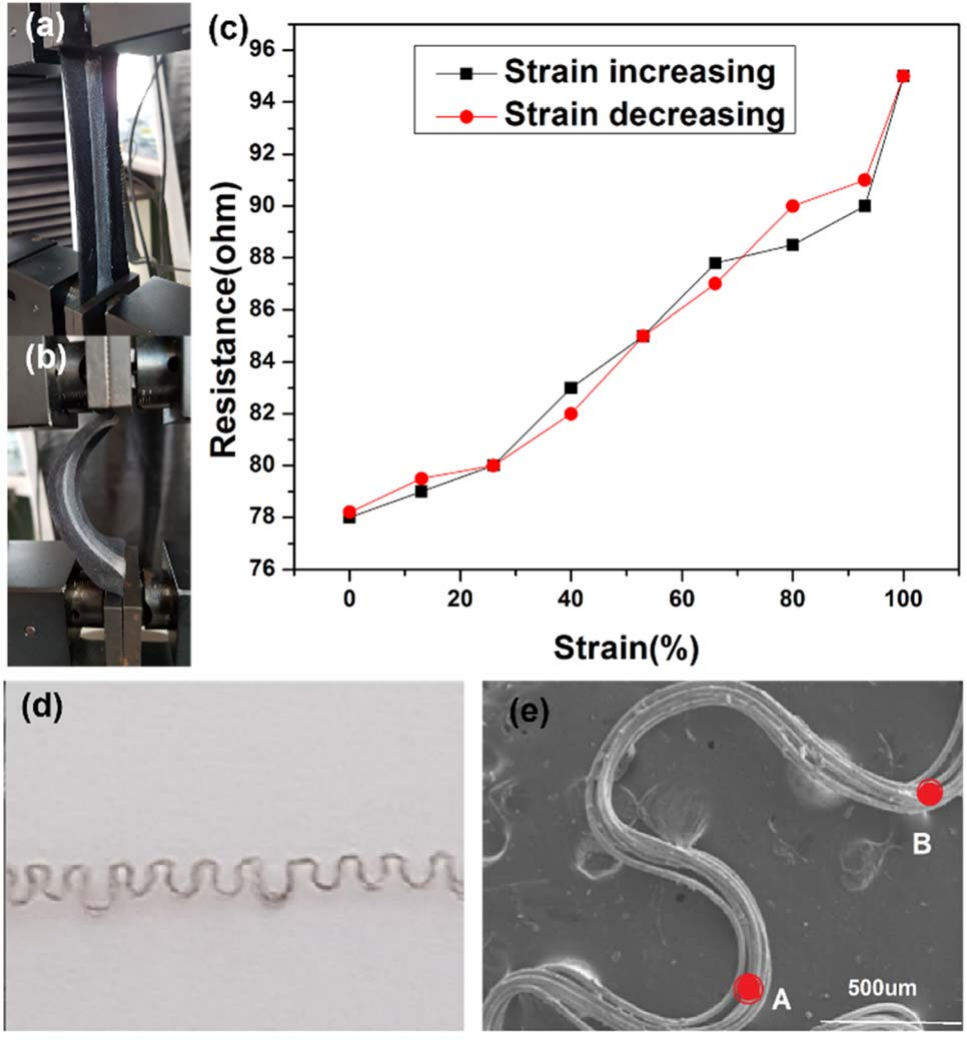
(a) Stretchable conductive device, upon stretching, (b) upon recovering using instron machine. (c) Resistance verses strain of the stretchable conductive device. (d) Image of a yarn unravelled from silver plated conductive fabric sample. (e) SEM image of a yarn unravelled from silver plated conductive fabric sample.

**Fig. 6:**
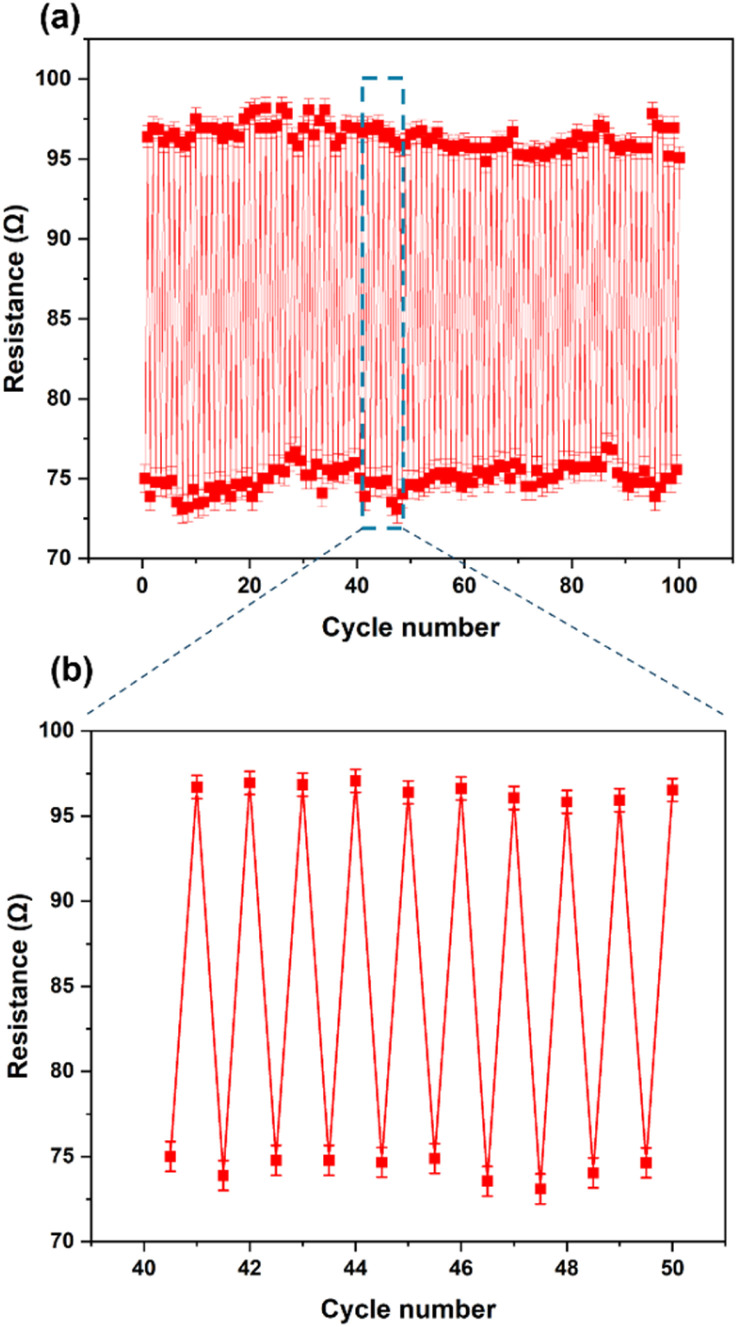
(a) Resistance at 0% and 100% strain for 100 cycles. (b) The magnified sensor response extracted from Fig. 5(a).

The prepared stretchable conductive device was placed between the two jaws of a tensile testing machine to evaluate its viability as a strain sensor. It was observed that when the device was stretched to a strain of 100%, its average resistance increased to 96.86 ± 0.67 Ω cm^−1^. Conversely, when the device was bent, as shown in Fig. 5a, the average resistance decreased to about 75.37 ± 0.88 Ω cm^−1^ (Fig. 5b). An increase in extension led to an increase in resistance between the two electrodes of the device (Fig. 5c). The resistance of the device changed upon stretching and recovery, due to disconnection and reconnection of bending points. The conductive yarn, which had been unravelled from the knitted fabric, is shown in Fig. 5d and e. The yarns in the stretchable device created bending points, such as point A and B, which connected and shortened the conductive path, resulting in a reduction in the device's resistance. More importantly, even after about 100 stretching cycles (Fig. 6a and b), the resistance changes repeatedly followed the device's stretch and recovery with high stability and low hysteresis (Fig. S3†). Therefore, multiple stretches did not compromise the functionality of the developed Ag coating. The gauge factor (ESI (S6)†) increased from 0.20 to 0.33 as the applied strain increased from 13% to 100%. Our results are summarized in Table S2.† Hence, our demonstration shows that stretchable conductive devices prepared using the developed Ag-coated yarns exhibit high reliability and repeatability.

## Conclusions

In this study, a novel, simple and universal approach for the preparation of a conductive silver layer on polyamide fibers through electroless plating is introduced. Initially, silver seeds are introduced by reducing Ag nanoparticles on fiber surface which is premetalized by Sn^2+^. Further reduction of Sn^2+^ is carried out by formaldehyde. Increasing the concentration of Ag^+^, leads to an increase in the conductivity of the fabric by formation of Ag/Ag_2_O layer with increasing particle size. This was proven clearly in SEM images, XPS, FTIR, UV-vis, XRD and TGA analysis. These yarns can be used to produce stretchable conductive devices which can be used for powering devices, *etc.* Furthermore, such conductive silver yarns have a potential of an array of promising applications in the field of smart textiles as biomedical industries, internet of things applications and flexible electronics.

Our conductive thread offers a unique combination of properties that make it superior to commercially available conductive yarns. Its low resistivity and stretchability make it ideal for use in stretchable electronic textiles, which have a range of applications in wearable electronics, medical monitoring devices, and other fields.

It is important to note that while our study demonstrated the successful use of silver nanoparticle-coated nylon fibers as conductive textiles, the potential for silver oxidation and redox currents in aqueous environments, such as body fluids or sweat, should be considered when evaluating their suitability for wearable applications. Further studies are needed to investigate the long-term stability and safety of using silver nanoparticle-coated nylon fibers in such applications.

## Author contributions

The manuscript was written through contributions of all authors. All authors have given approval to the final version of the manuscript.

## Conflicts of interest

The authors declare no conflict of interest.

## Supplementary Material

RA-013-D3RA01566A-s001
