# Supplementary material for: Electroless plating of premetalized polyamide fibers for stretchable conductive devices
Source: RSC Adv. 2023 Jun 20;13(27):18605–13. doi: 10.1039/d3ra01566a (PMC10280805; doi:10.1039/d3ra01566a)
Supplement: RA-013-D3RA01566A-s001 [file RA-013-D3RA01566A-s001.pdf]

Electronic Supplementary Material (ESI) for RSC Advances.
This journal is © The Royal Society of Chemistry 2023

Electroless plating of premetalized polyamide fibers for stretchable conductive devices

P. Vishakha T. Weerasinghe,[a] Ruchira N. Wijesena,*[b] Nadeeka D. Tissera,[b] Gayan Priyadarshana,d Nandula D. Wanasekara,[a] D.G. Kanchana Dissanayake[a] and K. M. Nalin de Silvac*[c]

*Corresponding author: ruchiraw@itum.mrt.ac.lk, kmnd@chem.cmb.ac.lk

a. Department of Textile and Clothing Textile and Clothing Technology, University of Moratuwa, Moratuwa, Sri Lanka.

b. Division of Textile and Clothing Technology, Institute of Technology, University of Moratuwa, Diyagama, Homagama.

c. Department of Chemistry, University of Colombo, Colombo 03, Sri Lanka.

d. Department of Engineering Technology, Faculty of Technology, University of Sri Jayewardenepura, Gangodawila, Nugegoda, Sri Lanka.

S1. Preparation of fabric samples

Nylon-6 filament yarn was knitted into a single jersey a circular fabric with a dimeter of 5cm, using circular lab knitting machine (Anytester, AT250F lab knitting machine). Then weighted fabric samples of 0.2 g were scoured to remove impurities utilizing 1 g/ml NaOH solution at 100^0C for 1 minute, then rinsed by a counter flow of distilled water.

S2. Fabrication of stretchable conductive device

A piece of silver plated yarn was layered on a stretchable fabric surface and two electrodes were attached to the two ends of the yarn. A of silicon rubber paste was printed on top of the yarn as shown in Fig. S1. Multiple print cure cycles were conducted to obtain 1mm thick layer of silicon.

The effect of stretching was observed by measuring the resistance of the device at variable percentages of displacements from the initial length. The strain was applied from 0-100% using a universal material testing machine (Instron 3365, USA). Resistance of the device for 100 stretch and recovery cycles was obtained by measuring the resistance of the device upon every stretch and recovery. Moreover, conductive stripes were knitted from silver plated yarns, elastomeric yarns and common nylon yarns by using a full computerized plain jacquard socks knitting machine(KJ-6F616, Zhuji Kejun Machinery Co., Ltd) and resistance per 4cm of the strip was measured.

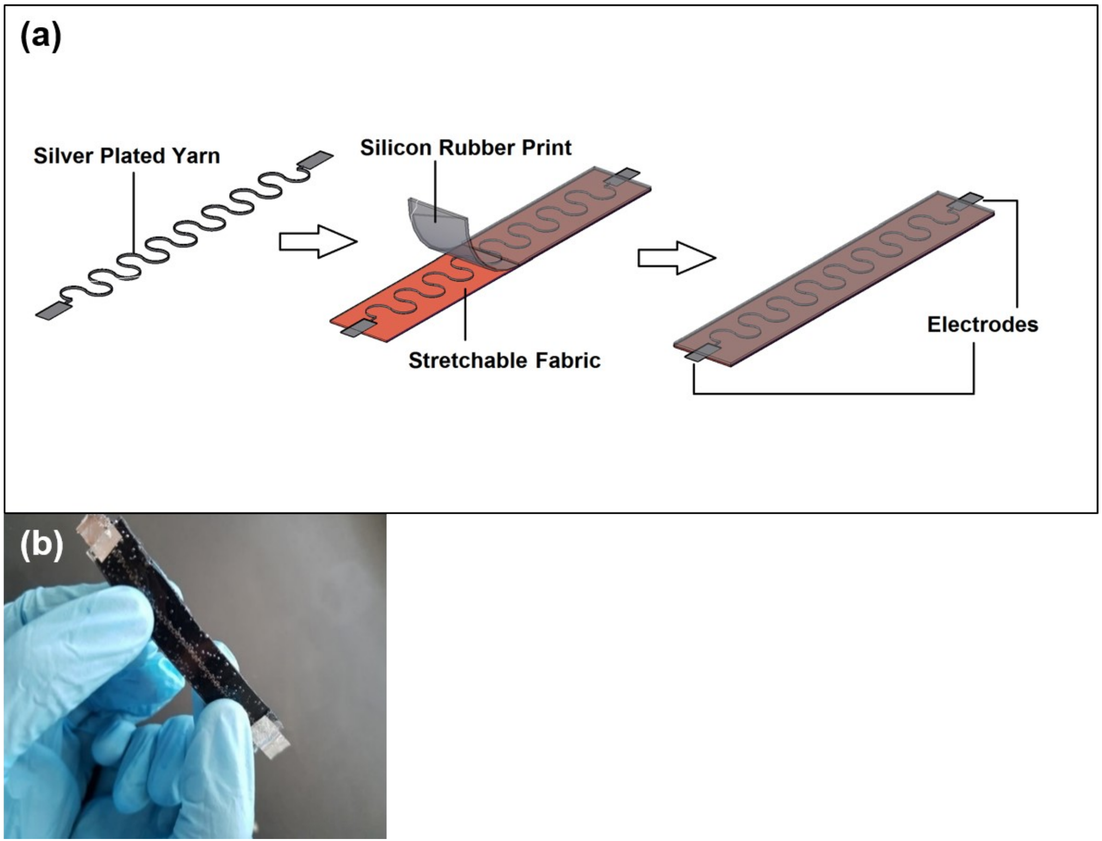

Fig. S1. (a) Schematic diagram of the fabrication process. (b) The device prepared by printing silicon layer on the yarn to stick yarn to the fabric and two electrodes are connected to ends of the print

S3. Polyamide hydrolysis

H^+ ions in the solution hydrolysis the polyamide and generate polar groups such as $–NH_2$ and -COOH on the fiber surface (Fig. S2). Further, the hydride irons bond with resulted $–NH_2$ and formed NH_3^+ end groups on the fiber surface (Eq. 1).

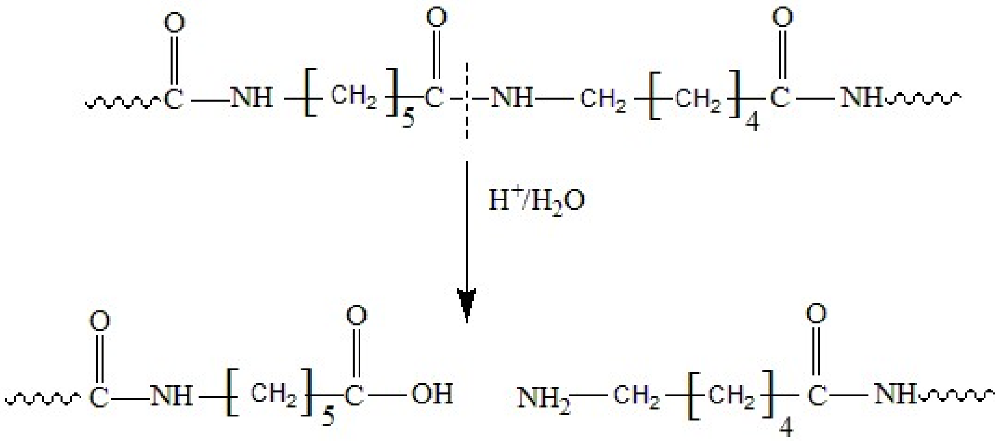

Fig. S2. Hydrolasis of polyamide in acid

$$CO - [NH_2 - (CH_2)_4 - CO] - N + H^+/H_2O \rightarrow NH_3^+ - (CH_2)_4 - COOH \quad (1)$$

S4. Silver reduction by Tollen's reagent

$$2AgNO_3 + 2NH_4OH = Ag_2O + 2NH_4NO_3 + H_2O \quad (2)$$

$$Ag_2O + 4NH_4OH = 2[Ag(NH_3)_2]OH + 3H_2O \quad (3)$$

$$[Ag(NH_2)_2]OH \xrightarrow{reduction\ regent} Ag + 2NH_3 + H_2O \quad (4)$$

S5. Silver nanoparticles

Comparatively, Ag 20% formed particles with larger sizes. As shown in Fig. S2 silver particle size of Ag 20% was less than 1 μm. Therefore, it can be claimed that particles formed by array of concentrations (Ag 2.5%, Ag 5%, Ag 10%, Ag 20%) are nano-size particles.

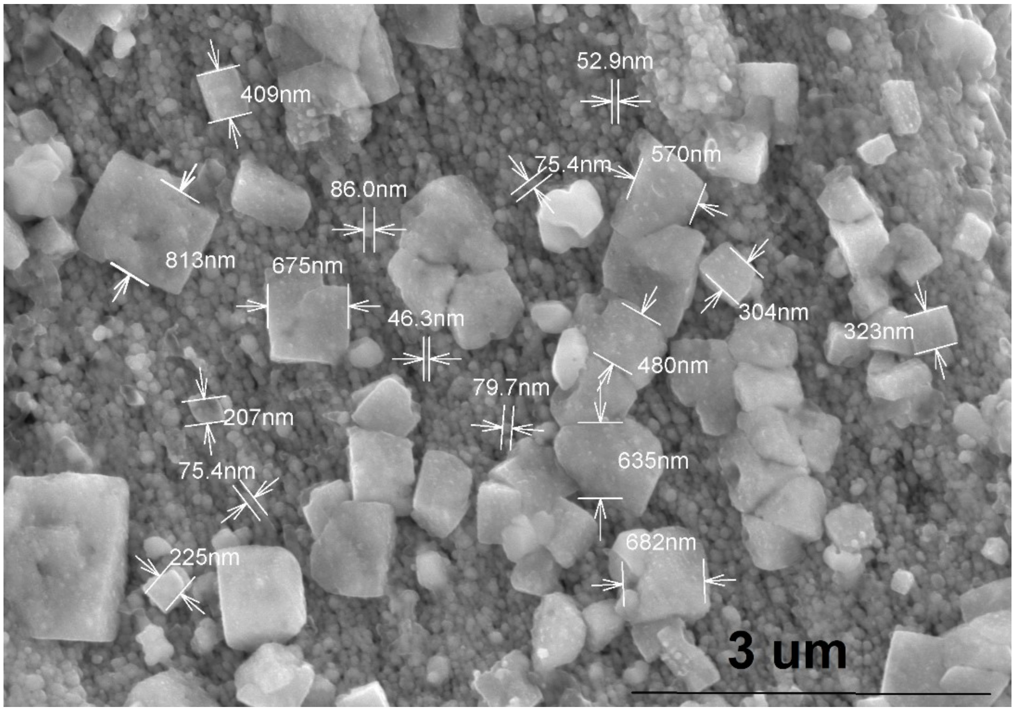

Fig S2. SEM image of Ag 20% with indicating the particle size

S6. TGA analysis results

Table S1. Ash content and weight loss data taken by TGA analysis.

**Yarn Sample**	**Ash content (%)**	**Weight loss (%)**

Polyamide	21.04	89.76
WFR	21.27	57.85
Ag 2.5%	22.48	58.48
Ag 5%	31.15	52.59
Ag 10%	43.96	37.80
Ag 20%	50.43	19.52

S6. Gauge Factor

The sensitivity of a strain sensor is generally evaluated with gauge factor (GF), which is the ratio of the percentage change of sensor response to the applied strain.

$$Gauge\ Factor\ (G.F.) = \frac{\Delta R/R_0}{\varepsilon} \quad (5)$$

where ΔR, R_0, and ε represent the change of resistance, the resistance at 0% strain, and the applied strain, respectively. The gauge factor increases from 0.20 to 0.33 in accordance with the increase of the applied strain from 13% to 100%.

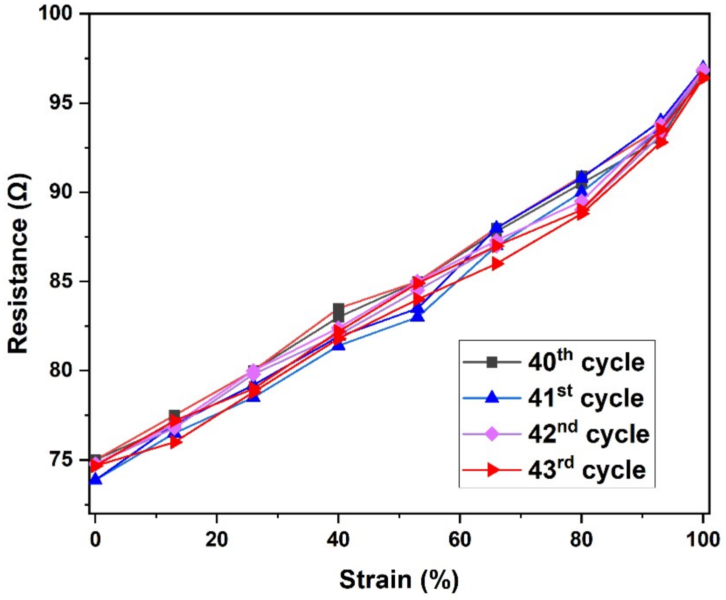

Fig S3. Hysteresis curves of the 40 th to 43rd stretch/release cycles.

S7. Hysteresis

S8. Comparison

Table S2. Comparison among stretchable conductive devices

Material	Fabrication Method	Stretchability	Gauge Factor
PDMS-CNT	Coating	100%	1.8 [1]
Plasticized PVC-CNT	Mixing of CNT with a substrate and sandwiching electrodes with an elastomer	100%	1.16 [2]
PDMS- carbon nanotubes and silver nanowires	Spin coat PDMS on CNT or Ag nanowire percolation networks	50%	0.05 [3]

Ag nanoparticle coated nylon - Silicon rubber	Sandwich the yarn between silicon rubber	100%	0.33 (This work)

References

1. Jin, C.; Liu, D.; Li, M.; Wang, Y. Application of highly stretchy PDMS-based sensing fibers for sensitive weavable strain sensors. *Journal of Materials Science: Materials in Electronics* **2020**, *31*, 4788-4796.
2. Dong, T.; Gu, Y.; Liu, T.; Pecht, M. Resistive and capacitive strain sensors based on customized compliant electrode: Comparison and their wearable applications. *Sensors and Actuators A: Physical* **2021**, *326*, 112720.
3. Cao, L.; Wang, Z.; Liu, Y.; Shi, R.; Wang, X.; Liu, J. A general strategy for high performance stretchable conductors based on carbon nanotubes and silver nanowires. *RSC advances* **2017**, *7*, 20167-20171.
